# Costs of accountable care organization participation for primary care providers: early stage results

**DOI:** 10.1186/s12913-016-1556-6

**Published:** 2016-07-28

**Authors:** Richard A. Hofler, Judith Ortiz

**Affiliations:** 1 Department of Economics, University of Central Florida, P.O. Box 161400, Orlando, FL 32816-1400 USA; 2 College of Health and Public Affairs, University of Central Florida, P.O. Box 162369, Orlando, FL 32816 USA

**Keywords:** Accountable care organizations, Primary care, Costs, Average treatment effects

## Abstract

**Background:**

Little is known about the impact of joining an Accountable Care Organization (ACO) on primary care provider organization’s costs. The purpose of this study was to determine whether joining an ACO is associated with an increase in a Rural Health Clinic’s (RHC’s) cost per visit.

**Methods:**

The analyses focused on cost per visit in 2012 and 2013 for RHCs that joined an ACO in 2012 and cost per visit in 2013 for RHCs that joined an ACO in 2013. The RHCs were located in nine states. Data were obtained from Medicare Cost Reports. The analysis was conducted taking a treatment effects approach where the treatment is joining an ACO. Propensity-score matching was employed to provide multiple single and pooled estimates of the average treatment effect on the treated.

**Results:**

Four-hundred thirty four to 544 RHCs (depending on the type of analysis and the variables used) were used in the several analyses. Seven of the RHCs joined an ACO in 2012 and 14 joined an ACO in 2013. The mean cost per visit for RHCs that did not join an ACO rose 4.40 % from 2011 to 2012 whereas the mean cost per visit for RHCs that joined an ACO rose by triple: 13.5 %. All of the pooled estimates of the average treatment effect on the treated from the propensity-score matching showed that joining an ACO was associated with higher mean cost per visit. The range of the estimated mean cost per visit differences was $17.19 (*p* value = 0.00) to $25.19 (*p* value = 0.00).

**Conclusions:**

This study is one of the first to describe the cost of ACO participation from the perspective of primary care provider organizations. It appears that for at least one type of primary care provider - the RHC - there are substantial costs associated with ACO participation during the first two years.

## Background

The Accountable Care Organization (ACO) is one of the new models of health care delivery that are intended to shift the provision of health services from an emphasis on volume to an emphasis on value. Whether the ACO model is successful or not will be assessed based on its achievement of two fundamental goals: 1) improvement in the quality of care and health outcomes of the population for which it is responsible, and 2) achievement of cost savings by a reduction in avoidable hospitalizations and emergency room visits via improving care coordination and preventive care services.

ACOs are composed of a range of health care organizations such as hospitals, groups of doctors, and/or other providers. In late 2011, CMS released regulations regarding provider participation in ACOs. These regulations included several provisions allowing primary care providers such as Rural Health Clinics (RHCs) to join Medicare Shared Savings Program (MSSP) ACOs, or to organize with other RHCs to become their own ACOs. Some of the RHCs that joined or formed MSSP ACOs were also selected to participate in the Advance Payment Model ACO, which was developed by CMS to meet the needs of smaller ACOs, such as those composed of physician-based and rural providers. These Advance Payment Model ACOs were awarded payments to support care coordination infrastructure. In this study we focus on Rural Health Clinics to determine the cost implications of ACO participation for rural primary care providers.

Recently, some findings of the impact of ACOs on costs per capita are beginning to emerge [1–3]. In addition, estimates of startup and first-year costs of forming an ACO have been made by both CMS and independent researchers (e.g., [4]). However, few studies examine the costs of a healthcare organization’s joining an ACO, and fewer still report on the costs associated with a *primary care organization’s* joining an ACO. To date, to the best of our knowledge, there have been no published findings on the costs to RHCs of ACO participation. Consequently, there is a strong need to investigate the costs incurred when primary care providers (organizations) such as RHCs join an ACO. Furthermore, there is an intriguing feature of cost per visit data for RHCs for the period 2007 through 2012 that we analyze here.^1^

We discovered that mean annual cost per visit for those RHCs that joined an ACO in 2012 jumped from 2011 to 2012 much more than for the other RHCs. Specifically, the rise for the ACO RHCs was bigger than for the others both in absolute terms ($15 per visit vs. $5 per visit) and in percentage term compared to the previous year (13.5 % higher than 2011 vs. 4.4 % higher than 2011.) See the Results section for more details.

These results raise a question: “*Could the bigger jump in cost per visit for the ACO RHCs be due to joining an ACO*?” In light of the above, the purpose of this study is to determine the relationship between ACO participation and cost per visit from the perspective of primary care providers. It is important to understand the cost of transitioning to and participating in ACOs from their perspective so that primary care providers may better plan for the future and remain viable in the evolving health care environment.

### Review of the literature

The transformation of our current volume-based payment system to one that is value-based will result in savings of $55 billion over 5 years for Medicare alone, according to CMS officials [5]. There are some indications that Medicare ACOs will achieve cost savings, although they may not do so initially. In their study of the Medicare Physician Group Practice (PGP) demonstration program (Medicare’s first physician pay-for-performance initiative), Pope et al. [6] determined that it is unlikely that Medicare ACOs will achieve large savings in the early stages of formation. Medicare beneficiaries aligned with the Pioneer Accountable Care Organization model were associated with smaller increases in total Medicare expenditures [7], or modest reductions in Medicare spending [8]. The Pioneer ACO program overall achieved a total savings of $87.6 million [9]. Elsewhere, Epstein et al. [10] compared patients cared for by Medicare ACOs to non-ACO patients, matched by geographic location, demographic, socioeconomic, and clinical characteristics. The results were lower inpatient, nonhospital and total costs for the ACO patients.

We know little about the initial and ongoing costs that *primary care* providers may incur should they choose to participate in ACOs. In fact, to the best of our knowledge, there have been no published findings on the costs to RHCs of ACO participation. The operating costs of providers that transform to Patient-Centered Medical Homes (PCMHs) may provide a hint of what providers can anticipate as they transition to become ACO participants. PCMHs share many of the same characteristics as ACOs, including patient-centeredness, population health management, care coordination, and the use of certified EHR technology.

Most studies of costs associated with PCMHs describe, rather than quantify, the costs of transitioning to PCMHs. Reiter et al. [11] describe costs as falling into two categories: costs of personnel time for transformation-related activities, and non-personnel resource costs such as for IT and supplies. Of the few studies that quantify costs, Nocon et al. [12] determined that Federally Qualified Health Centers that have a greater number of attributes associated with a PCMH had higher operating costs.

## Methods^2^

### Data sources and variables

The study population consisted of a panel of RHCs continuously operating during 2007–2013 as reported in the Center for Medicare and Medicaid Service’s (CMS’s) Online Survey, Certification and Reporting (OSCAR) database [13]. These RHCs were located in nine states: Alabama, California, Florida, Georgia, Kentucky, Mississippi, North Carolina, South Carolina, and Tennessee. Other sources of data were the Medicare Cost Reports [14, 15]. These data are publicly available through CMS (https://www.cms.gov/Research-Statistics-Data-and-Systems/Downloadable-Public-Use-Files/Cost-Reports/Hospital-2010-form.html; and https://www.cms.gov/Research-Statistics-Data-and-Systems/Downloadable-Public-Use-Files/Cost-Reports/HealthClinic.html). All investigators completed data management training regarding the protection of data and of human subjects. A data use agreement concerning the use of the Cost Report data was approved by the Centers for Medicare and Medicaid Services. Approval for the investigation was obtained through the Institutional Review Board of the University of Central Florida.

The study sample was composed of the 7 RHCs that joined MSSP ACOs in 2012, an additional 14 RHCs that joined in 2013, and the remaining RHCs that did not join an ACO. Among the total 21 RHCs in the sample were 11 that participated in Advance Payment Model ACOs.

The Medicare Cost Reports were the source of data for the study variables. The Medicare Cost Report is an annual cost report that Medicare-certified RHCs are required to submit to CMS or a Medicare Administrative Contractor. Although the cost report is limited in regard to some details of clinic operation, it is the most complete source of available data for RHCs at this time. The dependent variable was “cost per visit” (total cost of health care services + total nonreimbursable costs + total facility overhead)/ total visits provided to all patients by physicians, physician assistants, nurse practitioners, visiting nurses, and clinical social workers. The independent variables were: “TotFTE” (size of the RHC measured as the sum of the FTEs of physicians + physician assistants + nurse practitioners), “Provbsd” (a dummy variable where 1 = provider-based RHC and 0 = independent RHC), “Control” (classification of organizational control as for-profit, non-profit, or government), “Age” (number of years Medicare certified for participation in RHC program), and “Rural” (a dummy variable where 1 = RHC located in an isolated location and 0 = otherwise^3^) [16].

### Introduction: treatment effects estimation

We will be viewing participating in a Medicare Shared Savings Program ACO as a “treatment” and will, therefore, estimate the treatment effect of joining an ACO.

Treatment-effect estimators allow us to estimate three parameters. The potential-outcome means (POMs) are the means of *Y*_*1*_ (the potential outcome of a subject that did *not* receive treatment had it been treated) and *Y*_*0*_ (the outcome of a treated subject had it *not* received the treatment) in the population. The average treatment effect (ATE) is the mean of the difference (*Y*_*1*_ - *Y*_*0*_) for all subjects – those that received the treatment and those that did not. Finally, the average treatment effect on the treated (ATET) is the mean of the difference (*Y*_*1*_ - *Y*_*0*_) *among only the subjects that actually received the treatment*. We will report results for only the ATET for reasons described below in the Results section.

### Matching

Matching estimators are based on the idea of comparing the outcomes of subjects that are as similar as possible with the sole exception of their treatment status. One of the most widely-used methods to find comparable observations is propensity-score matching (PSM). PSM matches on the estimated predicted probabilities of treatment, known as the propensity scores.

### Finding multiple controls for each treated subject

Matching each treated subject with *one* other control subject is the most common behavior in most matching studies. However, you can match each treated subject with multiple control subjects with the opposite treatment level. It is important to be clear about the benefits and costs of multiple matching. Matching on more distant neighbors can reduce the variance of the estimator at a cost of a possible increase in bias. However, as you match more controls with each treated subject, you are finding successively poorer matches. Whether or not this becomes a problem (and if so, how big a problem) depends on how much worse the matches become as the number of matches per treated subject rises.

Our dataset contains three subsamples: (i) 2012 data for the 7 RHCs that joined an ACO in 2012, (ii) 2013 data for the 7 RHCs that joined an ACO in 2012, and (iii) 2013 data for the 14 RHCs that joined an ACO in 2013. We match each treated subject (an RHC that joined an ACO) with different numbers of control subjects (RHCs that did not join an ACO.) The number of matches (M) equals 1, 2, 3, 4, or 5.

We take three estimation paths. One involves separately estimating the effect of ACO participation on cost/visit for subsamples (i) and (ii) above. This permits us to determine whether or not the effect of ACO participation on cost/visit changed from 2012 to 2013. The second path estimates the treatment effect of ACO participation on the combined years of 2012 and 2013 for the 7 RHCs, a T = 2 panel. This allows us to account for unobserved time-constant confounders. Last, we estimate the treatment effect on subsample (iii) above, handling it as a single cross section.

There are five different ATET estimates in every one of the analyses covered in the previous paragraph. That is, we estimate ATET for M (number of controls matched with each treated RHC) = 1, then M = 2, then M = 3, then M = 4, and finally M = 5. One common way to learn the overall lesson from multiple studies is to use meta-analysis methods. We will regard each analysis that yielded an estimated ATET (one per value of M) as a “study” and use the Manzel-Haenszel (1959) [17] fixed effects method to uncover the general ATET on cost/visit of joining an ACO. Doing so has the advantages of employing a credible and widely-used method for combining multiple results and avoids making arbitrary decisions about which of the five estimates (M = 1, 2, 3, 4, 5) to report from each analysis.

## Results

In the Background section we mentioned the dissimilar increases in cost per visit for RHCs that joined an ACO in 2012 compared to those that did not. Table 1 shows both the mean and percentage change in cost per visit for the 7 RHCs that joined an ACO in 2012 and those RHCs that did not. Notice that cost-per-visit values are relatively stable for the ACO RHCs – until 2012. There is a jump in cost/visit in 2012 for both groups of RHCs from 2011 to 2012. However, the rise for the ACO RHCs is bigger than for the others both in absolute terms ($15 per visit vs. $5 per visit) and in percentage terms in two ways: compared to the mean cost per visit for 2007 through 2011 (9.0 % higher than the mean vs. 4.8 % higher) and compared to the previous year (13.5 % higher than 2011 vs. 4.4 % higher than 2011.) This motivates our analysis.

**Table 1:** Mean and percentage changes: cost/visit for the 7 RHCs that joined ACOs in 2012 and those that did not

	Cost per visit		Cost per visit	
	RHCs in ACO in 2012		RHCs not in ACO in 2012	
	mean	% change	mean	% change
2007	$119		$118	
2008	116	−2.50 %	107	−9.30 %
2009	116	0.00 %	116	8.40 %
2010	116	0.00 %	113	−2.60 %
2011	111	−4.30 %	114	0.90 %
2012	126	13.50 %	119	4.40 %

That analysis is accomplished in two steps.

**Step 1:** Estimate propensity scores

Propensity-score matching (PSM) matches on the predicted probabilities of treatment, known as the propensity scores. One estimates either a logit or probit model with the dependent variable being the treatment binary variable. In this case, the dependent variable is equal to 1 if the RHC joined an ACO and equals zero otherwise. The predicted probabilities of joining an ACO are the propensity scores in this study. Our logit model contains the major variables that the literature says will influence the decision to join an ACO *and* that we have in our dataset. Those variables are “size” of the RHC (as measured by the total FTEs, or full-time equivalents, for the RHC), and “rural” (as measured by the RUCA code^c^ for the RHC). Previous research has indicated that the size of physician practices is positively related to ACO participation [18], and that RHC size was positively related to an RHC’s willingness to join an ACO [19]. In regard to geographic location, RHCs located in isolated areas were 78 % less likely to be in ACOs than those located in areas with a RUCA classification of “urban” [19].

Table 2 contains the estimation results for the logit model from which propensity scores are estimated. Only the rurality variable appears to affect the likelihood that an RHC joins an ACO. It says that being in an isolated location reduces the likelihood that an RHC will join an ACO.

**Table 2:** Logit model used to generate propensity scores. Dependent variable is ACO Start12 = 1 if RHC joined an ACO in 2012 or in 2013

Predictor	β
Size (Total FTEs)	−0.388 (0.315)
Provider-based RHC	0.914 (0.400)
Control	−0.208 (0.469)
Age of RHC	0.0240 (0.734)
Rural	−2.928** (0.010)
Constant	−1.980 (0.204)
N	456
Pseudo R-sq	0.217
AIC	55.15
BIC	79.88

*p*-values in parentheses

** *p* < 0.05

**Step 2:** Estimate average treatment effect on the treated (ATET) using propensity-score matching

Recall from the Methods section that the average treatment effect (ATE) is the mean of the difference (*Y*_*1*_ - *Y*_*0*_) for all subjects – those that received the treatment and those that did not. The average treatment effect on the treated (ATET) is the mean of the difference (*Y*_*1*_ - *Y*_*0*_) among only the subjects that actually receive the treatment. We estimate the ATET for the following reasons:

The ATET tells us whether a particular treatment is beneficial only for those who were in the treatment group. RHCs and other providers participate in ACOs for several reasons. They evaluate not only the benefits of participating in an ACO such as possible improvement in patient outcomes, but also the drawbacks such as the start-up costs. In addition to such evaluation, a provider’s decision to become part of or form an ACO is inevitably influenced by the actions of its peers. RHC management are very interested in how their peers fare in this new ACO model. In other words, they are most interested in the experiences of the RHCs that participated in an ACO so they can decide about their RHC joining or forming an ACO. Thus, for this study that concerns strategic decisions of providers rather than clinical treatment decisions, we have chosen to estimate a treatment effects measure (the ATET) that shows how *only* those RHCs that participated in an ACO fared instead of ATE, which shows how *all* RHCs could have done (whether they were actually in an ACO or not).

Table 3 contains the results from the analyses of four subsamples. They are as follows:

**Table 3:** Estimated ATET by number of matches and pooled across the different numbers of matches. Year = 2013 and 7 RHCs that joined an ACO in 2012

Study	ATET	[95 % Conf. Interval]		% Weight
Part 1 (Year is 2012 and the 7 RHCs that joined an ACO in 2012, *n* = 544)				
Test of ATET = 0: z = 6.68, *p* = 0.000				
Matches = 1	26.740	8.022	45.458	15.61
Matches = 2	29.410	11.653	47.167	17.34
Matches = 3	26.610	8.931	44.289	17.50
Matches = 4	23.040	8.066	38.014	24.39
Matches = 5	22.430	7.691	37.169	25.17
Pooled ATET (note 1)	25.193	17.799	32.588	100.00
Percentage of mean	14.02 %			
Part 2 (Year is 2013 and the 7 RHCs that joined an ACO in 2012, *n* = 435)				
Test of ATET = 0: z = 3.17, *p* = 0.002				
Matches = 1	14.110	−10.488	38.708	18.81
Matches = 2	19.790	−5.592	45.172	17.67
Matches = 3	18.450	−5.030	41.930	20.65
Matches = 4	16.980	−6.285	40.245	21.03
Matches = 5	17.170	−5.664	40.004	21.83
Pooled ATET (note 1)	17.282	6.612	27.951	100.00
Percentage of mean	14.02 %			
Part 3 (Year is 2013 and the 14 RHCs that joined an ACO in 2013, *n* = 434)				
Test of ATET = 0: z = 3.73, *p* = 0.000				
Matches = 1	27.590	0.621	54.559	17.60
Matches = 2	23.840	−2.267	49.947	18.78
Matches = 3	20.180	−5.535	45.895	19.36
Matches = 4	17.740	−5.976	41.456	22.76
Matches = 5	19.850	−4.552	44.252	21.50
Pooled ATET (note 1)	21.545	10.231	32.859	100.00
Percentage of mean	17.48 %			
Part 4 (Panel for 2012 and 2013 and the 7 RHCs that joined an ACO in 2012, *n* = 984) Test of ATET = 0: z = 5.12, *p* = 0.000				
Matches = 1	13.490	−3.189	30.169	15.57
Matches = 2	17.020	3.300	30.740	23.02
Matches = 3	16.320	2.581	30.059	22.95
Matches = 4	19.670	4.441	34.899	18.68
Matches = 5	18.940	4.142	33.738	19.78
Pooled ATET (note 1)	17.185	10.603	23.766	100.00
Percentage of mean	14.05 %			

Note 1: Employing the meta-analysis fixed effect model using the method of Mantel and Haenszel, 1959 [19]

Note 2: Mean cost/visit = $121.43 (2012), $123.29 (2013), and $122.36 (2012 and 2013)

Note 3: Panel 4 shows the results of using the fixed effects model (the within transformation) for a T = 2 panel. This is not the same as the fixed effect model used in the Mantel-Haenszel method

i. Part 1: A single cross-section for 2012 and the 7 RHCs that joined an ACO in 2012
ii. Part 2: A single cross-section for 2013 and the 7 RHCs that joined an ACO in 2012
iii. Part 3: A single cross-section for 2013 and the 14 RHCs that joined an ACO in 2013
iv. Part 4: A T = 2 panel for 2012 and 2013 and the 7 RHCs that joined an ACO in 2012.

Most of the columns of each panel are self-explanatory. The column on the far right of each panel shows the contribution of each row (“study”) to the overall pooled ATET estimate.

The ATET estimates in Part 1 (2012) range from $22.42 (M = 5) to $29.41 (M = 2.) All of the estimated treatment effects of joining an ACO are significantly different from zero at the 5 % level or better. The pooled ATET estimate (combining all five of the estimates) is $25.19 and is significantly different from zero (z = 6.68 and *p* = 0.000.) The ATET estimates in Part 2 (2013) are uniformly lower than those in Part 1. All of the estimated ATET values are significantly different from zero at the 5 % level or better. The pooled ATET estimate is $17.28 (also lower than in Panel 1) and is significantly different from zero (z = 3.17 and *p* = 0.002).

Part 3 shows the results for the 14 RHCs that joined an ACO in 2013. Their pooled ATET estimate is lower than the first-year pooled ATET estimate for the RHCs that started in an ACO in 2012. It is $21.55 (vs. $25.19 for the 2012 RHCs) and is significantly different from zero (z = 3.73 and *p* = 0.000).

These results in Parts 1–3 suggest several points. First, joining an ACO does raise cost/visit. Second, the jump in cost can be substantial, with point estimates ranging from 14 % to nearly 21 %. Third, that increase lasts at least two years. Fourth, the rise seems to be less in the second year.

Finally, Part 4 contains the panel data (T = 2 years) estimates of the ATET for those RHCs that began in an ACO in 2012. We employed the fixed effects model (using the within transformation) for this short panel. The pooled ATET estimate is $17.18 and is significantly different from zero (z = 5.12 and *p* = 0.000).

Notice that the pooled ATET estimate for 2012 and 2013 combined (Table 3, Part 4) is $0.10 lower than the pooled ATET estimate for 2013 ($17.18 vs. $17.28) and is $8.01 lower than the pooled ATET estimate for 2012 ($17.18 vs. $25.19.) In other words, the estimate for 2012 and 2013 combined is lower than the lower estimate for either single year. This could be due to the fact that the method for estimating the single-year ATET values ignores time-constant unobserved factors (called unobserved heterogeneity or fixed effects or confounders) whereas the fixed effects estimator accounts for them. Time-constant (over 2012 and 2013) unobserved factors that might affect cost/visit include business goals of the clinic’s decision-makers,^4^ demographic factors of the community not captured in our data, and features of the local environment that can make doing business either cheaper or more costly. Evidently, these unobserved factors raised cost/visit during 2012 and 2013. Adjusting for them subtracts their impacts on cost/visit from the estimated ATET to give an estimated treatment effect that is closer to the true impact on cost/visit of joining an ACO.

## Discussion

This study was an examination of the impacts on costs of RHCs using a treatment effects approach where the treatment was joining an ACO. The results suggest several points. First, joining an ACO does raise cost/visit. Second, the jump in cost can be substantial, with point estimates ranging from 14 % to nearly 21 %. Third, that increase lasts at least two years. Fourth, the rise seems to be less in the second year.

While an investigation of the contributors to the change in costs is beyond the scope of this study, we can speculate about some of those factors. Although RHCs may receive a federal incentive grant to support starting an ACO, establishing and sustaining the necessary ACO infrastructure can be costly. In a recent study of the early experiences of RHCs that participate in ACOs, several reported that building the necessary infrastructure means added costs related to administration, electronic health record (EHR) system establishment and maintenance, and often, additional staff to meet new regulatory or quality expectations [20].

EHR systems are a fundamental feature of ACO organizational structure. By sharing patient data among the participant providers of the ACO, EHR is expected to improve patient quality of care and health outcomes. The initial costs of EHR implementation can be high, however, and even if ACO participants have EHR in place, their software systems may be incompatible.

Other costs are associated with important but labor-intensive activities such as obtaining patient consent to collect data. Once collected, Medicare ACOs require that the data be compiled, analyzed, and submitted. Several hours per month may be spent in such required committees as finance, quality care, and EHR.

Enforcing standards of quality for ACOs can also be costly. Medicare ACOs use 33 quality measures classified in four domains: patient experience, coordination of care and patient safety, preventative health, and caring for at-risk populations [21]. Additional costs for training and monitoring for quality improvement efforts can be incurred.

There are several policy considerations concerning the participation of RHCs and other primary care providers in ACOs. Needed are more clear guidelines regarding the expectations of primary care providers with limited administrative infrastructures such as those common to RHCs. Consideration must be given to the demographic makeup and limited clinical workforce of small rural communities. Although ACO design and participant requirements will continue to evolve, this model has many anticipated benefits to patients, providers, and the healthcare delivery system as a whole. Patients are expected to benefit from improved continuity of care and greater access to preventive care. Primary care providers could benefit from having greater access to capital for training, hiring or sharing staff, and developing technological infrastructure. Finally, ACOs are anticipated to reduce healthcare costs overall by reducing emergency department visits and hospitalizations.

### Limitations

Although the results uniformly show that joining an ACO does raise cost/visit, there are a few limitations to this study. First, although there exist several types of both Medicare and commercial ACOs in the current healthcare environment, our data were limited to RHCs participating in Medicare Shared Savings Program (MSSP) ACOs (which included Advance Payment Model ACOs). Second, the analyses were limited to RHCs in nine states only, and do not necessarily represent the changes in cost associated with ACO participation for RHCs in other areas of the U.S. Third, in selecting variables for the logit model to create a comparison sample for predicting ACO participation, we were limited to the available data. These data did not allow us to create other variables associated with ACO participation by physician practices, such as PCMH recognition [18]. Fourth, although several cost components were included, the specific cost components which may have contributed to the cost increase were not analyzed in this study. Finally, in part because it is early in the history of ACOs (the first MSSP ACOs were announced in January of 2012), the number of RHCs participating in ACOs is low. As a consequence, the analysis is based on a dataset that contains just twenty-one RHCs that joined an ACO. Keep in mind, though, that the smallest subsample had 434 observations (see Table 3) due to the large number of non-ACO RHCs in our dataset.

Despite these limitations, this study has value in describing changes in costs of some primary care providers during the early years of their participation in ACOs. From a practical standpoint, providers that are considering ACO participation need to be able to anticipate and budget for additional personnel, information technology, supplies-related expenses, and the like associated with the transition. The costs of preparing and maintaining the infrastructure necessary for ACO participation may diminish over time. It is also likely that the costs will vary depending on the length of time the provider has participated in an ACO. Additionally, this study has intentionally ignored the benefits during the first year of ACO participation. So, this study necessarily presents only part of the entire picture of the changes that can occur as a result of ACO participation.

## Conclusions

While it is early in ACO history to draw conclusions about the impact of ACO participation on the cost of healthcare per capita, this study is one of the first to describe the cost of ACO participation from the perspective of primary care provider organizations. It appears that for at least one type of primary care provider organization – the RHC – there are substantial costs associated with ACO participation during the first two years. Future studies will examine not only the costs related to ACO participation over a longer period, but also the factors that may contribute to those costs. Additional evidence is needed concerning the most successful ACOs that include rural providers, as well as those composed entirely of rural providers.

## Abbreviations

ACO, accountable care organization; ATE, average treatment effect; ATET, average treatment effect on the treated; CMS, center for medicare and medicaid services; EHR, electronic health record; MSSP, medicare shared savings program; PCMH, patient-centered medical home; PGP, physician group practice; POM, potential-outcome mean; PSM, propensity-score matching; RHC, rural health clinic

## Footnotes

1 We are investigating only those RHCs in Alabama, California, Florida, Georgia, Kentucky, Mississippi, North Carolina, South Carolina, and Tennessee. Seven RHCs in these states joined an ACO in 2012 and 14 joined in 2013, the most recent data that are available.

2 Some of this section contains parts of an introduction to treatment effects estimation contained in the Stata 13 documentation: “teffects intro—introduction to treatment effects for observational data” in the Stata documentation for the teffects command.

3 The Rural–urban Commuting Area (RUCA) Code has four categories: 1 = Urban, 2 = Large rural, 3 = Small rural, and 4 = Isolated. This binary variable “rural” equals one for each RHC with a RUCA code equal to 4. We chose this specification for this binary variable because our analysis of survey responses we received showed that RHCs in isolated areas are less likely to join ACOs.

4 These might include cost minimization, increasing market share, improving patient care at higher costs, etc.
